# Composite pheochromocytoma–ganglioneuroma secreting vasoactive intestinal peptide

**DOI:** 10.1530/EDM-25-0184

**Published:** 2026-10-01

**Authors:** Benjamin Brochu, Matthieu St-Jean, Yves Collin, Rabia Temmar, Frédéric Bernier, Karel Dandurand, Marie-Hélène Massicotte

**Affiliations:** ^1^ Division of Endocrinology, Department of Medicine, Centre Hospitalier Universitaire de Sherbrooke, Sherbrooke, Canada; ^2^ Department of Surgery, Centre Hospitalier Universitaire de Sherbrooke, Sherbrooke, Canada; ^3^ Department of Pathology, Centre Hospitalier Universitaire de Sherbrooke, Sherbrooke, Canada

**Keywords:** adrenal, endocrine cancers

## Abstract

**Summary:**

An 80-year-old female patient presented with a 10-year history of progressively worsening diarrhea and chronic hyponatremia. An abdominal computed tomography (CT) revealed a 6.8-centimeter right adrenal mass. A Ga-68 DOTATATE positron emission tomography (PET)/CT scan subsequently demonstrated a significant expression of somatostatin receptors on the tumoral shell (maximal SUV of 14.4), associated with a hypometabolic central portion. Endocrine investigation indicated elevated levels of both urinary and plasmatic normetanephrines. Given the watery diarrhea, VIP levels were measured, which were markedly elevated. Surgical resection confirmed a composite pheochromocytoma–ganglioneuroma with VIP immunoreactivity in both tumor components. In the postoperative period, the plasma levels of VIP and normetanephrines returned to normal with resolution of diarrhea, hyponatremia, and hypertension. This case illustrates a rare instance of composite pheochromocytoma–ganglioneuroma with co-secretion of catecholamines and VIP, with evidence of VIP production in both tumor components. The finding of VIP secretion in the pheochromocytoma and the ganglioneuroma suggests that both tumors might originate from the same cell of neural crest origin.

**Learning points:**

Pheochromocytomas are frequently associated with excessive catecholamine production and can rarely co-secrete VIP, leading to secretory diarrhea and electrolyte disturbances, such as hypokalemia and hyponatremia, while pheochromocytomas usually lead to constipation.
Chronic watery diarrhea refractory to standard treatments should prompt evaluation for rare neuroendocrine tumors, such as VIP-secreting pheochromocytomas or composite adrenal tumors, especially when imaging reveals adrenal masses.
Surgical removal of composite pheochromocytoma–ganglioneuroma with VIP co-secretion can lead to complete resolution of diarrhea, normalization of electrolytes, and stabilization of blood pressure, underscoring surgery as definitive therapy.

## Background

Chronic secretory diarrhea is a common clinical challenge with a broad differential diagnosis ranging from infectious and inflammatory conditions to functional disorders. However, persistent watery diarrhea refractory to standard therapies should prompt evaluation for rare neuroendocrine etiologies, including vasoactive intestinal peptide (VIP)-secreting tumors known as VIPomas (1). While the majority of VIPomas originate from pancreatic tissue, a minority of cases arise from extra-pancreatic sites, such as the adrenal medulla.

Pheochromocytomas are typically characterized by catecholamine hypersecretion, presenting with hypertension, palpitations, and episodic symptoms. Only rarely do these tumors exhibit hormone co-secretion beyond catecholamines, and reports of VIP-producing pheochromocytomas are exceedingly uncommon. The simultaneous presence of VIP secretion in both pheochromocytoma and ganglioneuroma components in a composite adrenal tumor with documented VIP secretion is even more exceptional with only a previous case described in the literature to date.

This report describes a unique case of a composite pheochromocytoma–ganglioneuroma in an elderly patient presenting with chronic secretory diarrhea due to VIP co-secretion and documents resolution of symptoms following surgical excision.

## Case presentation

An 80-year-old Caucasian female with a history of dyslipidemia and osteoporosis was evaluated for a long-standing history of severe and progressively worsening diarrhea since 2012 (see Fig. 1 for clinical timeline). She reported experiencing up to eight episodes of watery stools daily, often accompanied by mucus. Her symptoms persisted despite trials of multiple therapies, including eluxadoline, psyllium, loperamide, and cholestyramine. Colonoscopic examination and histological analysis of colonic biopsies ruled out infectious and inflammatory etiologies. Further investigations were unremarkable, with normal anti-transglutaminase antibodies and normal serum tryptase. Fecal calprotectin was undetectable, arguing against intestinal inflammation. Stool examination for parasites was negative, and bacterial cultures for Salmonella, Shigella, Yersinia enterocolitica, Escherichia coli O157:H7, and Campylobacter were also negative. Finally, testing for Whipple’s disease by PCR on blood was negative. Her diarrhea was therefore initially attributed to a functional disorder. Partial relief of the diarrhea could be achieved with a combination of loperamide taken 1–8 times per day, along with cholestyramine once per day. Chronic hyponatremia was also documented from 2017 onward with serum sodium levels consistently ranging between 128 and 132 mmol/L without causative medication. A subsequent workup confirmed a state of hypoosmolar hyponatremia. Investigations revealed a serum sodium level of 123 mmol/L, a serum osmolality of 260 mmol/kg H_2_O, and an inappropriately concentrated urine osmolality of 476 mmol/kg H_2_O, with a urine sodium level below 20 mmol/L. Clinical assessment indicated a low-normal volume status. Similar findings were observed on another occasion, with a serum sodium level of 128 mmol/L, a serum osmolality of 272 mmol/kg H_2_O, and a urine osmolality of 377 mmol/kg H_2_O. Additionally, she exhibited mild hypertension with an average blood pressure of 145/90 mmHg.

**Figure 1:**
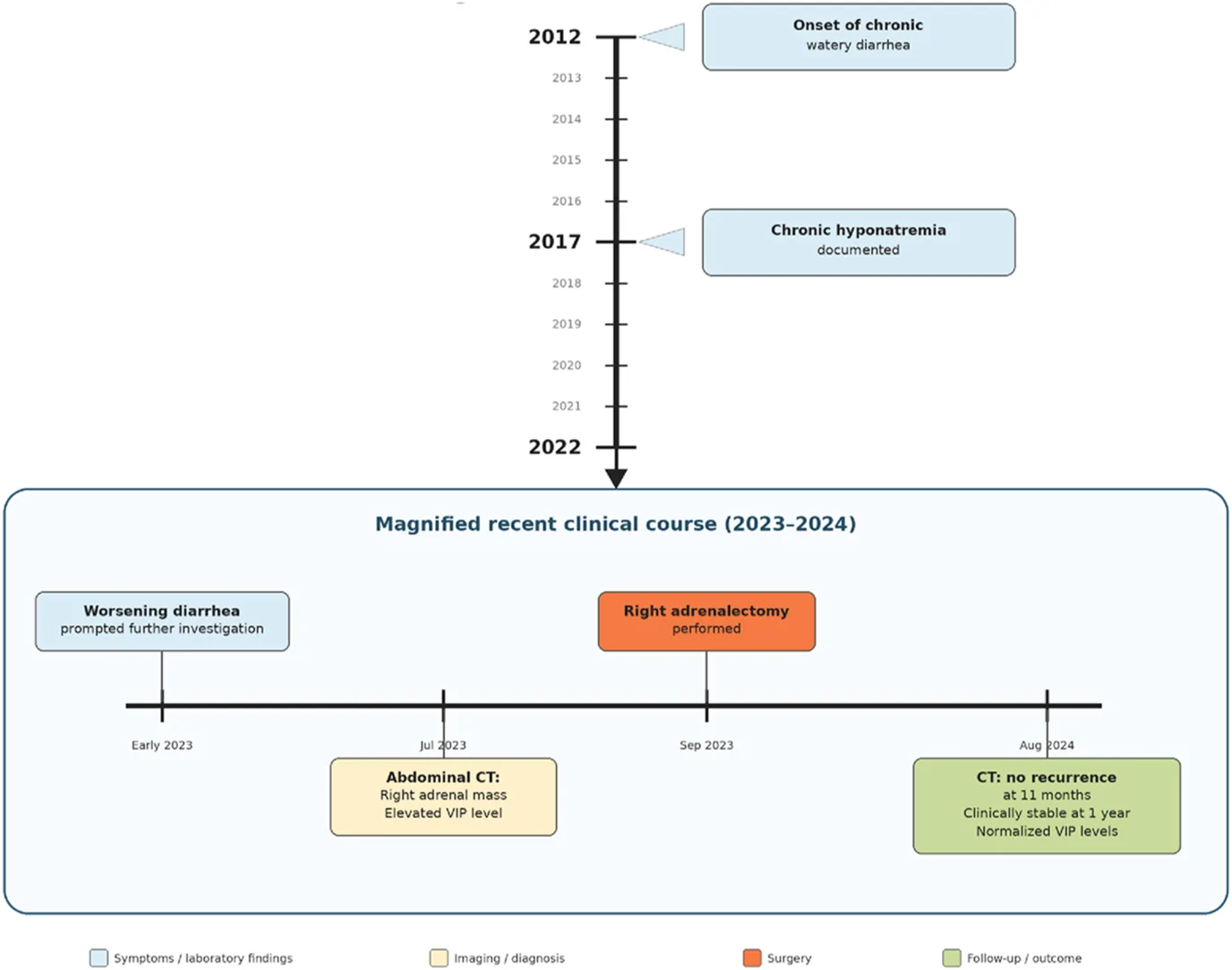
Clinical timeline.

In early 2023, her diarrhea worsened to approximately 8 bowel movements per day despite treatment. In this context, an abdominal CT scan was completed and revealed a right adrenal mass measuring 68 × 44 × 50 mm with an unenhanced density of 19 Hounsfield units (HU).

The patient exhibited no clinical manifestations consistent with Cushing’s syndrome, nor did she report frequent occurrences of headache, palpitations, diaphoresis, nervousness, or tremors.

## Investigation

Endocrine evaluation showed a significant elevation in both 24-h urinary normetanephrines (696–763 nmol/day, approximately 3.5 times the upper limit of the normal) and plasma normetanephrines (see Table 1). The 1-mg dexamethasone suppression test was slightly above the normal at 61 nmol/L (normal: <50 nmol/L), with normal salivary and 24-h urinary cortisol levels. Aldosterone-to-renin ratio was normal at 40 pmol × L/ng × L, with plasma aldosterone concentration at 201 pmol/L and direct renin concentration at 5 ng/L. Notably, due to the unusual persistent severe diarrhea in the context of the potential normetanephrine-secreting pheochromocytoma, the serum vasoactive intestinal peptide (VIP) level was measured and found to be nearly three times above the upper limit of normal. Both serum calcitonin and urinary 5-HIAA were within normal ranges.

**Table 1:** Endocrine evaluation at baseline. Abnormal values are in bold.

Test	Initial value	Normal range
24-h urinary metanephrines		
Dopamine (nmol/day)	1,848	0–2,612
3-Methoxytyramine (nmol/day)	353	0–370
Normetanephrine (nmol/day)	**763**	0–192
Metanephrine (nmol/day)	96	0–169
Other laboratory findings		
Sodium (mmol/L)	**130**	135–145
Potassium (mmol/L)	3.9	3.5–5
Plasma normetanephrines (nmol/L)	**2.97**	0–1.2
Plasma metanephrine (nmol/L)	0.23	0–0.48
Serum VIP		
pg/mL	**210**	0–78
pmol/L	**87**	0–32.4
Calcitonin (pg/mL)		
pg/mL	<1	0–10
pmol/L	<0.23	0–2.3
24-h urinary 5-HIAA (μmol/day)	17.7	0–42
Cortisol* (nmol/L)	61	<50
Plasma aldosterone concentration (pmol/L)	201	<350
Direct renin concentration (nmol/L)	5	3–16

5-HIAA, 5-hydroxyindoleacetic acid.

* Overnight 1-mg dexamethasone suppression test.

A Ga-68 DOTATATE PET/CT displayed significant overexpression of somatostatin receptors on the outer tumoral shell with a maximal SUV of 14.4, associated with a hypometabolic central portion with a SUV of 1.4 (see Fig. 2). The lesion showed higher uptake than the liver, which had a maximal SUV of 9.3. A subsequent FDG PET/CT demonstrated low metabolic activity of the adrenal mass (maximum SUV of 2.7). Since the tumor center had a positive FDG expression, it was hypothesized that the lesion had a tissular component to it and was not merely central necrosis. Neither the Ga-68 DOTATATE PET/CT nor the FDG PET/CT showed distant lesions.

**Figure 2:**
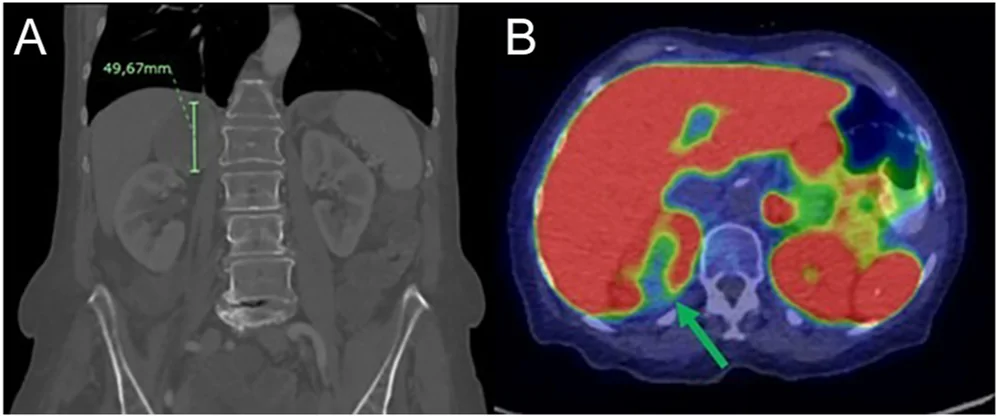
(A) Abdominal CT scan demonstrating the 68 × 44 × 50 mm adrenal mass. (B) Ga-68 DOTATATE PET/CT demonstrating significant uptake of the tumoral shell (see the arrow).

A provisional diagnosis of pheochromocytoma with co-secretion of VIP and catecholamines was established.

## Treatment

Doxazosin was initiated and titrated up to 6 mg twice daily to achieve adequate blood pressure control as part of optimal preoperative management. Given the size of the adrenal mass, the patient subsequently underwent open right adrenalectomy. The procedure was performed without complications.

Pathological assessment of the resected mass revealed a composite tumor consisting of regions of pheochromocytoma and ganglioneuroma. The Ki-67 proliferation index was less than 1% in both regions. The tumor had a PASS (Pheochromocytoma of the Adrenal Gland Scaled Score) of 3, based on diffuse architecture (2 points) and marked nuclear pleomorphism (1 point), indicating low malignant potential. Immunohistochemical analysis revealed diffuse and strong VIP immunoreactivity within both the pheochromocytoma and ganglioneuroma components (see Fig. 3). While the staining was markedly positive in both areas, a formal quantitative assessment of intensity and distribution was not performed.

**Figure 3:**
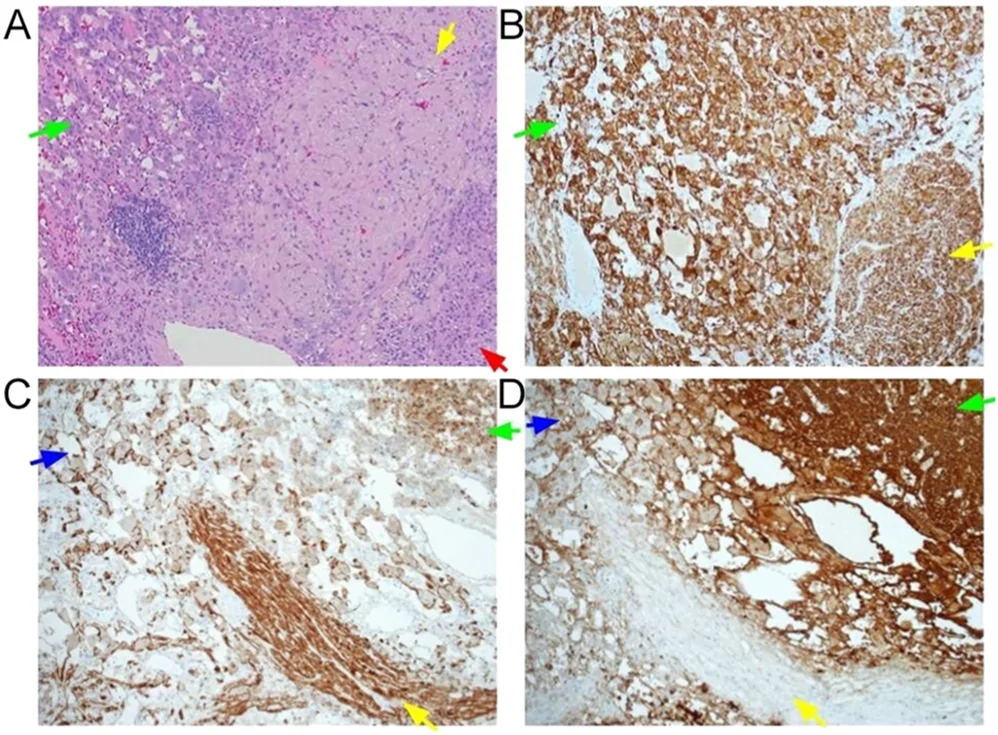
(A) Hematoxylin and eosin (H&E) staining (10X) demonstrating a composite adrenal tumor with distinct interface between the pheochromocytoma (left, green arrow) and ganglioneuroma (central, yellow arrow) components, alongside adjacent nonneoplastic adrenal cortex (lower right corner, red arrow). (B) Vasoactive intestinal peptide (VIP) immunohistochemistry (10X) showing strong cytoplasmic immunoreactivity within both the pheochromocytoma (left, green arrow) and ganglioneuroma (right, yellow arrow) elements. (C) S-100 protein immunohistochemistry (10X) highlighting the central ganglioneuroma component (center, yellow arrow) demarcated by the surrounding pheochromocytoma (green arrow) with its sustentacular cells within the pheochromocytoma (blue arrow). (D) Chromogranin A immunohistochemistry (10X) displaying diffuse positivity in the pheochromocytoma component (right, green arrow) and its sustentacular network (left, blue arrow) enclosing the central ganglioneuroma (center, yellow arrow).

A genetic evaluation was conducted using a panel to detect known pheochromocytoma and paraganglioma-associated mutations. The panel included 11 genes (fumarate hydratase (FH), MAX, NF1, RET, SDHA, SDHAF2, SDHB, SDHC, SDHD, TMEM127, and VHL genes). However, no mutation was identified. Although the 11-gene panel covers the most well-established susceptibility genes associated with pheochromocytoma, the absence of a pathogenic variant does not entirely rule out hereditary predisposition. Current panels do not capture all possible germline or mosaic alterations, nor do they exclude novel, unidentified susceptibility genes.

## Outcome and follow-up

Following surgery, the patient’s diarrhea and hyponatremia resolved, and her blood pressure and urinary normetanephrines normalized. At one-year follow-up, she reported only mild occasional diarrhea, and laboratory values for sodium, metanephrines, and VIP all remained within normal limits. At 11 months following surgery, abdominal computed tomography showed no signs of tumor recurrence.

## Discussion

Pheochromocytomas are neuroendocrine tumors originating from the adrenal medulla typically characterized by the secretion of catecholamines. However, in rare instances, these tumors can produce ectopic hormones, such as adrenocorticotropic hormone (ACTH), corticotropin-releasing hormone (CRH), testosterone, aldosterone, and VIP (1). VIP is a peptide hormone primarily secreted by the gastrointestinal tract, where it promotes the secretion of water and electrolytes into the intestinal lumen (2). Excessive VIP secretion, most commonly associated with pancreatic neuroendocrine tumors, can lead to the watery diarrhea, hypokalemia, and achlorhydria (WDHA) syndrome. In contrast, pheochromocytoma is usually associated with constipation. Therefore, in cases of pheochromocytoma accompanied by significant diarrhea, there is a need to consider the possibility of VIP co-secretion. Furthermore, the vasodilatory properties of VIP can counteract the hypertensive effects typically induced by catecholamine excess, potentially masking the classic clinical presentation of pheochromocytoma and delaying diagnosis (2).

The association between vasoactive intestinal peptide excess and chronic hyponatremia is atypical, as the hallmark of VIPoma remains the WDHA syndrome. While hyponatremia is not a classic component of this syndrome, its presence in this patient is likely multifactorial. Chronic gastrointestinal fluid losses as those experienced by our patient can induce a state of effective circulating volume depletion, triggering a non-osmotic release of arginine vasopressin, which promotes renal-free water retention and accounts for the inappropriately high urine osmolality relative to serum osmolality. Excess VIP may also directly contribute through other unknown mechanisms, as VIP receptors are known to be present in both the kidneys and the central nervous system (3). With the correction of hyponatremia following the surgical resection of the tumor, it can be hypothesized that the resolution of VIP hypersecretion eliminated the driving force behind both the secretory diarrhea and the associated neuroendocrine disruptions, which likely allowed for the restoration of normal fluid and electrolyte homeostasis.

Concerning the result of the 1-mg overnight dexamethasone suppression test (ODST) that was mildly abnormal at 61 nmol/L (normal < 50 nmol/L), it can be described using current terminology as possible mild autonomous cortisol secretion. However, clinically significant cortisol excess was not supported by the absence of Cushingoid features, normal salivary cortisol, normal 24-h urinary cortisol, and the overall biochemical and clinical context. Furthermore, non-suppression after dexamethasone is a recognized phenomenon in pheochromocytoma, and recent large cohort data indicate that post-ODST non-suppressed cortisol is common (4). The proposed mechanisms include catecholamine-driven paracrine stimulation of the zona fasciculata and mild hypothalamic–pituitary–adrenal axis activation, suggesting that non-suppressed cortisol in pheochromocytoma should not necessarily be interpreted as definitive evidence of autonomous cortisol secretion (4).

Composite pheochromocytomas are an uncommon entity, featuring both pheochromocytoma and neural components within the same adrenal tumor. The most common neural component (70% of cases) is ganglioneuroma, a benign tumor generally originating from autonomous ganglion cells (5). The ganglioneuroma component is generally nonsecretory. Coexistence of both tumor types in a composite tumor might be explained by their common origin from neural crest cells. To our knowledge, only four case reports in the literature have mentioned a composite pheochromocytoma co-secreting VIP (2, 5, 6, 7), including one malignant pheochromocytoma (6). An overview of these cases is provided in Table 2.

**Table 2:** Reported cases of VIP-secreting composite pheochromocytoma–ganglioneuroma with clinical presentation, biochemical profile, pathological features, and outcomes.

Case report	Clinical presentation	Biochemical findings	Pathological characteristics	Outcome
Yee *et al.* (2)	64-year-old male with hypertensive emergency and the WDHA syndrome	Elevated metanephrine and VIP levels	Composite pheochromocytoma; VIP immunostaining in neoplastic cells, without mention of specific tumoral components	Surgical resection resolved symptoms and normalized laboratory values
Kikuchi *et al.* (5)	12-year-old female with initial headache and hypertension and the WDHA syndrome appearing later at age 25	Elevated catecholamines initially; later, serum VIP increased when diarrhea developed	Composite pheochromocytoma–ganglioneuroma; both components were VIP-positive	The patient ultimately died of metastatic disease
Onozawa *et al.* (6)	47-year-old female with general muscle weakness, watery diarrhea, and neurofibromatosis type 1	Elevated catecholamines and VIP	Composite pheochromocytoma with ganglioneuroma; VIP staining restricted to the ganglioneuroma component	Symptoms and signs subsided after surgical removal of the tumor
Ende *et al.* (7)	45-year-old female with persistent secretory diarrhea for 6 years and then muscular weakness and myalgia from hypokalemic rhabdomyolysis	Elevated catecholamines and VIP	Composite pheochromocytoma with negative VIP staining	Diarrhea stopped after surgery, with normalization of laboratory parameters

Of these, only one case demonstrated VIP immunoreactivity in both tumor components (6). Our case represents the second such report, highlighting the possibility of a shared neural crest progenitor cell capable of differentiating into both chromaffin and neural elements with secretory capabilities. The mechanisms underlying VIP production in both tumor components remain unclear but may reflect several nonexclusive processes. One possibility is differentiation from a common neural crest-derived progenitor retaining multipotent capacity, allowing expression of neuroendocrine features in both chromaffin and ganglionic cells. Alternatively, paracrine or microenvironmental interactions between pheochromocytoma and ganglioneuroma elements might promote cross-induction of neuropeptide synthesis, thereby explaining VIP expression in both compartments. Concerning the results of the Ga-68 DOTATATE PET/CT, two distinct tumoral zones were observed, one showing higher expression of somatostatin receptors on the outer shell of the tumor and another displaying lower expression in the central region. Variations in somatostatin receptor (SSTR) expression, particularly among the different neuroendocrine components, could explain this heterogeneous uptake pattern. This may then reflect the differing levels of DOTATATE uptake between the pheochromocytoma and ganglioneuroma components of the tumor.

Surgical excision remains the definitive treatment as evidenced by the resolution of diarrhea, normalization of electrolytes, and stabilization of blood pressure following tumor removal in this patient.

This case underscores the importance of considering VIP co-secretion in patients with pheochromocytoma or composite adrenal tumors who present with chronic secretory diarrhea and electrolyte disturbances. The demonstration of VIP immunoreactivity in both the pheochromocytoma and ganglioneuroma components lends support to, though does not definitively prove, the theory of a common neural crest origin and highlights the diverse secretory capabilities of these rare neoplasms.

Early recognition and surgical management are essential as complete resection can lead to full resolution of symptoms and normalization of biochemical abnormalities. This report adds to the limited literature on composite pheochromocytoma–ganglioneuroma with VIP co-secretion and emphasizes the need for awareness of atypical presentations in pheochromocytomas.

## Declaration of interest

The authors declare that there is no conflict of interest that could be perceived as prejudicing the impartiality of the work reported.

## Funding

This research did not receive any specific grant from any funding agency in the public, commercial, or not-for-profit sector.

## Patient consent

Written informed consent for publication of their clinical details and/or clinical images was obtained from the patient.

## Author contribution statement

BB wrote the manuscript and was involved in the diagnosis and management the patient. MS-J wrote and edited the manuscript. M-HM, KD, and FB edited the manuscript and were involved in the diagnosis and management of the patient. RT was involved in histopathology section and prepared histology images. YC was responsible for the patient’s surgery. All authors reviewed and approved the final draft. This case report has been prepared in accordance with the CARE guidelines, and a CARE checklist has been completed.
